# Glucose tolerance status is a better predictor of diabetes and cardiovascular outcomes than metabolic syndrome: a prospective cohort study

**DOI:** 10.1186/1758-5996-4-25

**Published:** 2012-06-08

**Authors:** Camila Furtado de Souza, Mériane Boeira Dalzochio, Francisco Jorge Arsego de Oliveira, Jorge Luiz Gross, Cristiane Bauermann Leitão

**Affiliations:** 1 Primary Care, Universidade Federal do Rio Grande do Sul, Porto Alegre, Brasil; 2 Endocrine Division of Hospital de Clínicas de Porto Alegre, Universidade Federal do Rio Grande do Sul, Rua Ramiro Barcelos 2350, Prédio 12, 4° andar, 90035-003, Porto Alegre, RS, Brazil

**Keywords:** impaired fasting glucose, impaired glucose tolerance, type 2 diabetes, metabolic syndrome, cardiovascular disease

## Abstract

**Backround:**

To evaluate the importance of oral glucose tolerance test (OGTT) in predicting diabetes and cardiovascular disease in patients with and without Metabolic Syndrome from a population treated in a primary care unit.

**Research design and methods:**

A prospective cohort study was conducted with subjects regularly attending the primary care unit of Hospital de Clínicas de Porto Alegre. Participants underwent a 75 g OGTT. Metabolic syndrome definition was based on the criteria of IDF/AHA/NHLBI-2010.

**Results:**

Participants mean age was 61 ± 12 years (males: 38%; whites: 67%). Of the 148 subjects included, 127 (86%) were followed for 36 ± 14 months, 21 (14%) were lost. Subjects were classified into four groups based on baseline OGTT: 29% normal (n = 43), 28% impaired fasting glucose (IFG; n = 42), 26% impaired glucose tolerance (IGT; n = 38), and 17% diabetes (n = 25). Metabolic syndrome prevalence was lower in normal group (28%), intermediate in IFG (62%) and IGT (65%) groups, and higher among subjects with diabetes (92%; P <0.001). Incidence of diabetes increased along with the stages of glucose metabolism disturbance (normal: 0%, IFG: 16%, IGT: 28%; P = 0.004). No patient with normal OGTT developed diabetes, regardless metabolic syndrome presence. Diabetes at baseline was the major determinant of cardiovascular disease occurrence (normal: 0%, IFG: 4%, IGT: 0%, diabetes: 24%; P = 0.001). In Cox-regression analysis, only the 2 h OGTT results were associated with diabetes (OR = 1.03; 95%CI 1.01–1.06; P <0.001) and cardiovascular disease development (OR = 1.013; 95%CI 1.002–1.025; P = 0.024).

**Conclusions:**

In this sample of subjects undergoing diabetes screening, the OGTT predicted diabetes and cardiovascular disease more effectively than the metabolic syndrome status.

## Background

Hyperglycemia is a well-known risk factor for micro- and macrovascular disease [1] and is associated with increased morbidity and mortality [2,3]. Alterations on glucose homeostasis have been described preceding diabetes mellitus (DM) diagnosis, and are known as “prediabetes”. Prediabetes comprises two subcategories, impaired fasting glucose (IFG) and impaired glucose tolerance (IGT), classified based on glucose levels at fasting and after a glucose challenge (oral glucose tolerance test; OGTT) [4]. Both conditions are associated with increased risk for DM [5,6]. Prediabetes is a risk factor for cardiovascular events [7] and, recently, IGT has been associated with microvascular disease, retinopathy and microalbuminuria, conditions traditionally attributed to DM [8-13].

Metabolic Syndrome (MetS), a cluster of cardiovascular risk factors characterized by insulin resistance, abdominal obesity, dyslipidemia and hypertension, is associated with coronary heart disease, leading to increased cardiovascular and total mortality [14,15]. Patients with type 2 DM have a higher prevalence of MetS (85% vs. 24% in general population) [16,17] and the aggregation of MetS components amplifies the risk for micro- and macrovascular complications [17].

Because both prediabetes and MetS are risk factors for DM and cardiovascular disease (CVD), it is likely that the two conditions coexist in the same individuals. However, only a few studies have evaluated this association [18,19]. In addition, it is not known if the categorization of the patients based on OGTT results would predict DM and CVD development better than the presence of MetS.

Therefore, the aim of this study was to evaluate the importance of OGTT results in predicting DM and CVD development in patients with and without MetS from a population at risk for type 2 DM treated in a primary care unit.

## Research design and methods

### Patients

A prospective cohort study was performed with 148 patients with abnormal fasting plasma glucose (FPG) values (100 – 125 mg/dl), during a DM screening test. The criteria to participate in the screening was based on American Diabetes Association (ADA) recommendations: age ≥45 years old, body mass index (BMI) ≥25 kg/m^2^, hypertension, dyslipidemia, polycystic ovarian syndrome, family history of type 2 DM in a 1^st^ degree relative, previous personal history of gestational DM or fetal macrosomia, IFG or IGT in previous testing, and history of cardiovascular disease [20]. All subjects underwent an OGTT at baseline with 75 g of glucose; fasting and 2 h plasma glucose levels were measured. Subjects were evaluated by the researchers twice in the Primary Care Unit Santa Cecília/Hospital de Clínicas de Porto Alegre: first in the period between January and December 2005; and then between January and February 2010 patient’s charts were reevaluated to identify those who progressed to DM and/or developed CVD. The study protocol was approved by the Research Ethics Committee of Hospital de Clínicas de Porto Alegre.

### Baseline assessments

Clinical (age, gender, ethnicity, family history of type 2 DM, history of hypertension and blood pressure levels, smoking and physical activity), anthropometrical (weight, height, and waist circumference), and laboratorial data (screening fasting glucose, OGTT: fasting and 2 h post-challenge glucose levels, creatinine and lipid profile) were recorded from patients charts. The office blood pressure was measured with an aneroid sphygmomanometer with the patient in a sitting position, after a 5-min rest. The waist circumference was measured at the midpoint between the iliac crest and the lower costal edge, and the BMI (weight/height^2^) was also calculated [21]. The 10-year cardiovascular risk was estimated by Framingham risk score [22].

### Definitions

The glucose status was classified at baseline, and 4 categories were created according to fasting and 2 h OGTT results: normal (FPG <100 mg/dl and 2 h glucose <140 mg/dL), IFG (FPG between 100 and 125 mg/dl, and 2 h glucose <140 mg/dL), IGT (FPG ≤125 mg/dL and 2 h glucose between 140 and 199 mg/dL) and DM (FPG ≥126 mg/dL or 2 h glucose ≥200 mg/dL) [20]. Patients in the normal group had an abnormal fasting plasma glucose during the screening (values between 100 and 125 mg/dL), but it was not confirmed by the OGTT results.

Definition of MetS was based on the unified criteria of International Diabetes Federation/American Heart Association/National Heart, Lung, and Blood Institute [23]. Patients with 3 or more of the following factors were considered with MetS: fasting glucose ≥100 mg/dL (or drug treatment for diabetes), triglycerides levels ≥150 mg/dL (or drug treatment for elevated triglycerides), HDL cholesterol level <40 mg/dL in men and <50 mg/dL in women (or drug treatment for low HDL), systolic blood pressure (SBP) ≥130 mmHg and/or diastolic blood pressure (DBP) ≥85 mmHg (or antihypertensive drug treatment), and waist circumference >94 cm in men and >80 cm in women.

The development of DM was defined as: FPG ≥126 mg/dL or 2-h plasma glucose ≥200 mg/dL during an OGTT or a random plasma glucose ≥200 mg/dL in the presence of classic symptoms of hyperglycemia or hyperglycemic crisis [20]. In the absence of unequivocal hyperglycemia, criteria 1 and 2 were confirmed by repeat testing. The cardiovascular outcome was considered the combination of cardiovascular death, nonfatal myocardial infarction, nonfatal stroke, congestive heart failure, angina with documented myocardial ischemia, and non-traumatic lower limb amputation.

### Laboratory methods

Fasting and 2 h glucose levels were measured by hexokinase method. Serum creatinine was measured by Jaffe method, and lipid profile by enzymatic colorimetric method. LDL-cholesterol was calculated using Friedewald's equation, for samples with triglycerides levels of <400 mg/dL [24].

### Statistical analysis

Continuous variables are presented as means ± standard deviation and median (interquartile interval), and categorical as absolute (number) and relative frequency (percentage). Student *t* test was used to compare continuous variables. Variables with non-normal distribution were log transformed. Chi-square test, with residual analysis, was used to compare categorical variables. One-way analysis of variance (ANOVA) with Tukey's post-hoc test was used for continuous variables. Kaplan-Meier curves (Log-Rank test) were used to assess the risk of development of DM and CVD and Cox’s regression analysis was employed to adjust the results to variables related to DM and CVD pathogenesis. A P value <0.05 (two-tailed) was considered significant. This sample had >90% of power to detect differences in risk for DM and was not powered for CVD development.

## Results

### Baseline characteristics

A total of 148 patients [men: n = 57 (38%); white: n = 99 (67%)] were included. According to OGTT results, 29% (n = 43) of the patients had normal blood sugar, 28% (n = 42) had IFG, 26% (n = 38) had IGT (8 isolated IGT; 30 IGT combined with IFG) and 17% (n = 26) had DM.

Clinical and laboratory characteristics of patients, according to OGTT categories, are presented in Tables 1 and 2, respectively. No differences regarding age, gender, ethnicity, family history of type 2 DM, smoking and sedentarism, as well as lipid profile and creatinine levels, were found among groups. Hypertension prevalence was higher in subjects with DM (88.0% vs. 47%, P = 0.003), and BMI values were higher in IGT group (31.2 ± 5.7 vs. 25.7 ± 2.5 kg/m^2^, P = 0.019), in comparison with the normal group. Fasting plasma glucose values increased along with the categories of impaired glucose metabolism (normal: 92 ± 5; IFG: 109 ± 6; IGT: 108 ± 11, and DM: 123 ± 15 mg/dl; P <0.001), but this variable could not differentiate the two subcategories of prediabetes.

**Table 1:** Clinical characteristics of patients according to oral glucose tolerance test groups

	**Normal (n = 43)**	**IFG**^**£**^**(n = 42)**	**IGT**^**£**^**(n = 38)**	**DM**^**£**^**(n = 25)**	**P**
**Age (years)**	59 ± 12	61 ± 13	61 ± 11	64 ± 11	0.50
**Men – n (%)**	21 (49)	17 (41)	10 (26)	9 (36)	0.22
**White – n (%)**	27 (63)	29 (69)	25 (66)	18 (72)	0.64
**Smoking - n (%)**	10 (23)	6 (14)	2 (5)	3 (12)	0.12
**Hypertension - n (%)**	20 (47)*	30 (73)	22 (58)	22 (88)*	0.003
**Systolic BP**^**§**^**(mmHg)**	135 ± 23	146 ± 25	137 ± 17	146 ± 19	0.055
**Diastolic BP**^**§**^**(mmHg)**	83 ± 13	87 ± 13	87 ± 10	89 ± 10	0.12
**Family History of DM - n (%)**	10 (23)	8 (19)	9 (24)	5 (20)	0.91
**Sedentary – n (%)**	20 (47)	24 (57)	26 (68)	16 (64)	0.48
**Cardiovascular Risk score**^**ǂ**^	8 (5–15)	9 (3–13)	6 (2–12)	10 (2–15)	0,41
**BMI**^**†**^**(kg/m2)**	25.7 ± 2.5*	29.6 ± 4.6	31.2 ± 5.7*	29.2 ± 5.1	0.019
**Waist Circumference (cm)**					
**Men**	98.1 ± 8.2	103.0 ± 8.2	97.6 ± 7.7	98.3 ± 3.9	0.36
**Women**	94.4 ± 11.9	96.6 ± 8.6	100.6 ± 9.4	102.0 ± 12.2	0.23

Data expressed in mean ± standard deviation, number of cases (%) and median (interquartile interval).

^£^Oral glucose tolerance test groups: IFG impaired fasting glucose, IGT impaired glucose tolerance, DM diabetes mellitus.

^§^BP blood pressure, ^†^BMI body mass index, ^ǂ^ Framingham Score: cardiovascular risk in ten years.

*Difference between groups.

**Table 2:** Laboratory characteristics of patients according to oral glucose tolerance test groups

	**Normal (n = 43)**	**IFG**^**£**^**(n = 42)**	**IGT**^**£**^**(n = 38)**	**DM**^**£**^**(n = 25)**	**p**
**Fasting Plasma Glucose (mg/dL)**	92 ± 5*	109 ± 6	108 ± 11	123 ± 15*	0.001
**2 hour Plasma Glucose (mg/dL)**	96 ± 24*	117 ± 16*	161 ± 17*	239 ± 37*	<0.001
**Total Cholesterol (mg/dL)**	209 ± 43	218 ± 47	203 ± 33	209 ± 49	0.51
**HDL- Cholesterol (mg/dL)**	49 ± 11	52 ± 13	47 ± 11	46 ± 10	0.18
**Triglycerides (mg/dL)**	164 ± 172	151 ± 68	174 ± 99	195 ± 104	0.52
**LDL- Cholesterol (mg/dL)**	130 ± 35	135 ± 43	123 ± 29	124 ± 44	0.48
**Creatinine (mg/dL)**	0.96 ± 0.17	0.96 ± 0.24	0.94 ± 0.17	0.96 ± 0.31	0.97

Data expressed in mean ± standard deviation.

^£^Oral glucose tolerance test groups: IFG impaired fasting glucose, IGT impaired glucose tolerance, DM diabetes mellitus.

*Difference between groups.

All groups had comparable and low cardiovascular risk scores [normal: 8 (5–15) vs. IFG: 9 (3–13) vs. IGT: 6 (2–12) vs. DM: 10 (2–15)%/10 years; P = 0.41]. In opposition, the prevalence of MetS was lower in the normal glucose group, similar in the two classes of prediabetes, and higher in DM group [normal: n = 12 (28%); IFG: n = 26 (62%); IGT: n = 24 (65%) and DM: n = 24 (92%), P <0.001].

### Follow-up results

Patients were followed for an average of 37 ± 14 months, and 21 subjects were lost from follow-up (14%). One-hundred patients without DM and 110 without CVD at baseline completed the study, providing data for DM and CVD incidence, respectively. Lifestyle modifications were recommended for all patients with DM, 91% (n = 29) of those with IFG, and 94% (n = 30) of those with IGT. Metformin was used for DM prevention in 3% (n = 1) of patients with IFG and 12% (n = 4) of those with IGT.

**Figure 1:**
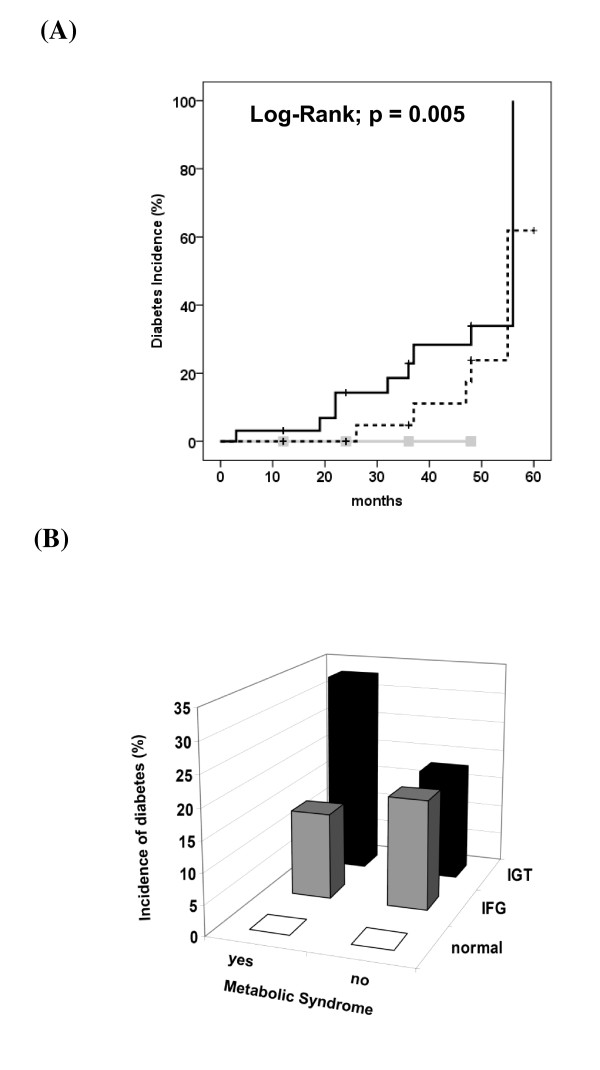
Diabetes incidence according to (A) oral glucose tolerance test groups (Normal: solid gray line with squares; IFG: dashed black line; IGT: solid black line); Log-Rank; P = 0.005 for normal vs. IFG and IGT; and (B) to the combination of Metabolic Syndrome presence and glucose status; Chi-squared; MetS present: P = 0.018 for normal and IFG vs. IGT and MetS absent: P = 0.045 for normal vs. IFG and IGT. IFG = impaired fasting glucose, IGT = impaired glucose tolerance.

No patient with normal OGTT developed DM during the observational period, while 5 (16%) subjects with IFG and 9 (28%) with IGT did (Figure 1A; P log-rank = 0.005; with statistical differences among all groups in the chi-squared residual analysis). As expected, individuals with the MetS had a higher incidence of DM, in comparison with those without (with MetS: 19% vs. without MetS: 8.5%; P log-rank = 0.032). However, the glucose status was a better predictor of DM development (Figure 1B), since no patient with normal OGTT developed DM, regardless the presence of MetS. Interestingly, in patients without MetS, the incidence of DM was comparable in both groups of prediabetes (normal: 0%, IFG: 18% and IGT: 18%; P = 0.045), while in those with MetS the incidence was higher in the IGT group (normal: 0%, IFG: 14% and IGT: 33%; P = 0.018). In Cox-regression analysis, FPG (OR = 1.08; 95%CI 1.02–1.14; p <0.001) and 2 h plasma glucose (OR = 1.04; 95%CI 1.02–1.06; p <0.001) were associated with the development of DM, even after adjustment for age, ethnicity and waist circumference or BMI. When fasting and 2 h plasma glucose were included in the same model simultaneously, only 2 h plasma glucose remained associated with DM development (OR = 1.03; 95%CI 1.01–1.06; p <0.001). Similarly when fasting and 2-hour plasma glucose were adjusted for individual MetS components, only the 2 hour value was associated with DM development. As metformin is known to decrease DM incidence, patients on this medication during the follow-up were excluded from analysis and no modifications was observed in results (data not shown).

**Figure 2:**
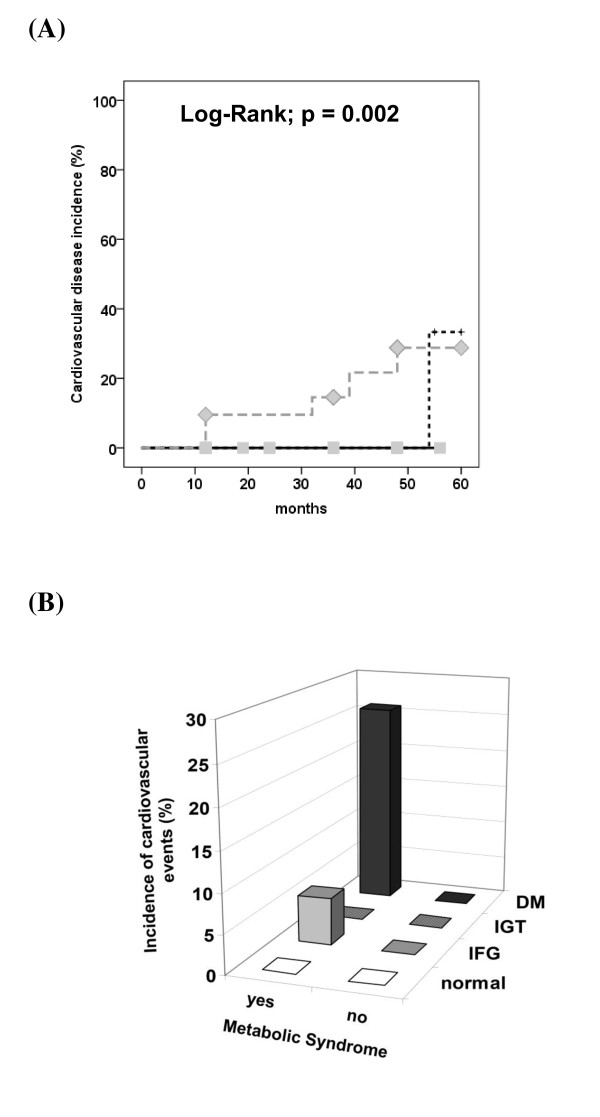
Cardiovascular incidence according to (A) oral glucose tolerance test groups (Normal: solid gray line with squares; IFG: dashed black line; IGT: solid black line; DM: dashed gray line with diamonds); Log-Rank; P = 0.002 for normal, IFG, IGT vs. DM; and (B) to the combination of Metabolic Syndrome presence and glucose status; Chi-squared; MetS present: P = 0.022 for normal and IFG and IGT vs. DM; MetS absent: no patient developed CVD. IFG = impaired fasting glucose, IGT = impaired glucose tolerance, DM = Diabetes Mellitus, CVD = cardiovascular disease.

Four percent of the patients (n = 6) developed CVD during the follow up. The incidence of CVD was significantly higher among patients with DM at baseline (normal: 0%, IFG: 4%, IGT: 0% and DM: 24%; P <0.001) (Figure 2A). As for DM incidence, MetS was a predictor of CVD development (with MetS: 9.5% vs. without MetS: 0%; P Log Rank = 0.027), and it seems that this association can be explained by the coexistence of DM at baseline OGTT (Figure 2B). In the multivariate analysis, only the 2 h plasma glucose values were associated with CVD (OR = 1.013; 95%CI 1.002–1.025; P = 0.024), while the FPG lost its association, after adjustments for age, gender, presence of hypertension, total cholesterol and smoking, or for MetS components.

## Discussion

In this sample of subjects from a primary care unit undergoing DM screening, the glucose status categorization based on OGTT results was a better predictor for the development of DM than the presence of MetS. The 2 hour value seemed more important than the fasting one in determining DM incidence and in predicting CVD.

Both IFG and IGT were risk factors for DM, as it has been reported in previous studies [5,6]. In our cohort, the DM incidence was higher in subjects with IGT in comparison with those with IFG, probably because the majority of subjects belonging to IGT had also IFG, since a screening FPG between 100 and 125 mg/dl was the inclusion criterion of the study. The coexistence of both glucose abnormalities has been formerly described as a stronger risk factor for DM development than each condition isolated [6]. Nevertheless, based on our results and others [25,26] the post-glucose challenge values seem to be more relevant than the fasting values in predicting DM.

Regarding CVD development, DM was the major risk factor, as it has been already well established [27-29]. In our study, participants who developed CVD had both DM and MetS. Liu et al. also found that increased CVD risk in individuals with IFG or diabetes was largely driven by the coexistence of multiple metabolic disorders rather than hyperglycemia per se [30]. Nevertheless, Hadaegh et al. described that MetS did not add to DM to predict incident CVD in Iranian population [31].

Recently, a meta-analysis showed that IFG and IGT are also associated with a mild increment in the risk for CVD [7]. We could not confirm these findings, since our study was not powered to detect CVD incidence differences. Our results are in accordance with DECODE [25] study, in which the 2 h glucose was a better predictor for CVD than fasting glucose.

Our results confirm the higher prevalence of MetS in prediabetes individuals in comparison to those with normal glucose values reported in Japanese and German samples [18,19]. However, in both studies prediabetes was considered as a unique group, and this is the first study reporting the MetS prevalence in separate strata of prediabetes (IFG and IGT). On the other hand, the prevalence of MetS in the DM group from the present cohort was high, and similar to previous studies conducted in patients with clinically recognized type 2 DM [17]. This finding was unexpected, since the subjects included in this study had mild and early DM, diagnosed based on OGTT results, and a lower cardiovascular risk profile, in comparison with patients with well-established DM.

The presence of MetS was also associated with DM development, but the glucose status identified individuals at higher risk more efficiently, since no patient, despite the MetS status, developed DM if their OGTT values were normal. These results are in accordance to previous studies who described that diagnosis of the MetS did not confer increased risk for incident diabetes, independent of its individual components [32]. Thus, the use of OGTT correctly predicts DM development, and were more efficient than categorization based on MetS presence. Similarly to the results for DM incidence, MetS was a predictor of CVD, though DM diagnosis accounted for almost all the risk observed.

Our results bring up back the discussion about the relevance of the MetS in predicting future cardiovascular events. There is still controversy whether the aggregation of MetS components confers a higher risk for CVD than the simple summation of each risk factor [33]. In the current study, the MetS diagnosis was not better than just one of its components, the glucose tolerance status, in predicting DM and CVD development. Thus, the clinical utility of MetS diagnosis in this scenario can be questioned, and clinical emphasis should be on treating effectively any CVD risk factor that is present [34]. Also, to prevent the metabolic syndrome or prediabetes and its consequences, these risk factors should be diagnosed and treated early, and this study demonstrates that individuals from a population at risk for having an abnormal glucose metabolism can be predicted, by simple clinical and laboratorial parameters which are available in a primary care setting.

The sample size and the low CVD risk profile of the included patients limited the evaluation of factors associated with the incidence of CVD. Moreover, the low number of patients with isolated IGT precludes the comparison of this group with those with isolated IFG. The HbA1c values were not available in the beginning of the study, since the use of HbA1c levels for screening or predicting DM was not routine in our center during that time [35].

## Conclusion

In conclusion, although the prevalence of MetS increases along with the severity of glucose homeostasis impairment, the OGTT results, with emphasis in the 2 h post-challenge values, are the major determinants of DM and CVD development. Our findings reinforce the importance of the OGTT in stratifying the risk for DM and CVD development, in patients with or without MetS.

## Abbreviations

AHA, American Heart Association; ADA, American Diabetes Association; ANOVA, One- way analysis of variance; BMI, Body mass index; CI, Confidence interval; CVD, Cardiovascular Disease; DBP, Diastolic Blood Pressure; DCCT, Diabetes Control and Complications Trial; DECODE, Diabetes Epidemiology Collaborative analysis of Diagnostic criteria in Europe; DM, Diabetes Mellitus; FPG, Fasting Plasma Glucose; HbA1c, Glycated hemoglobin; IDF, International Diabetes Federation; IFG, Impaired Fasting Glucose; IGT, Impaired Glucose Tolerance; MetS, Metabolic Syndrome; NHLBI, National Heart, Lung, and Blood Institute; OGTT, Oral Glucose Tolerance Test; OR, Odds ratio; SBP, Systolic Blood Pressure.

## Competing interests

The authors declare that they have no competing interests.

## Authors’ contributions

CFS, MBD, CBL contributes in execution, analysis, manuscript drafting and critical discussion. FJAO, JLG contributes in study design, analysis, manuscript drafting and critical discussion. All authors read and approved the final manuscript.
